# Longitudinal inconsistencies in women’s self-reports of lifetime experience of physical and sexual IPV: evidence from the MAISHA trial and follow-on study in North-western Tanzania

**DOI:** 10.1186/s12905-022-01697-y

**Published:** 2022-04-15

**Authors:** Tanya Abramsky, Sheila Harvey, Neema Mosha, Grace Mtolela, Andrew Gibbs, Gerry Mshana, Shelley Lees, Saidi Kapiga, Heidi Stöckl

**Affiliations:** 1 Department of Global Health and Development, London School of Hygiene and Tropical Medicine, 15-17 Tavistock Place, London, WC1H 9SH UK; 2 Mwanza Intervention Trials Unit, PO Box 11936, Mwanza, Tanzania; 3 Gender and Health Research Unit, South African Medical Research Council, 491 Peter Mokaba Road, Durban, South Africa; 4 Centre for Rural Health, School of Nursing and Public Health, University of KwaZulu-Natal, Durban, South Africa; 5 National Institute for Medical Research, Isamilo Road, Mwanza, Tanzania; 6 Department of Infectious Diseases Epidemiology, London School of Hygiene and Tropical Medicine, Keppel Street, London, WC1E 7HT UK; 7 Institute for Medical Information Processing, Biometry, and Epidemiology, Ludwig-Maximilians-Universität München, Elisabeth-Winterhalter-Weg 6, 81377 München, Germany; 8 Pettenkofer School of Public Health, Elisabeth-Winterhalter-Weg 6, 81377 München, Germany

**Keywords:** Intimate partner violence, Measure, Scale, Reliability, Inconsistency, Longitudinal, Tanzania

## Abstract

**Background:**

Intimate partner violence (IPV) against women is pervasive throughout the world, with profound consequences for women’s health. Research to understand the extent, causes and consequences of IPV relies on self-reported data on violence, and yet there is a paucity of research into the consistency with which women report lifetime IPV over time.

**Methods:**

We use data from the control group of the cluster randomised trial and a follow-on longitudinal study in Tanzania to examine discrepancies in women’s reported experience of lifetime physical IPV and sexual IPV over three time-points (T0, T29, T53 months). Among those reporting lifetime history of IPV at T0, we calculate the proportion who subsequently report no lifetime history at T29 and/or T53 (‘discrepant’ reporting). We use logistic regression to explore associations between discrepant reporting and respondent baseline characteristics, the nature of their IPV experiences at baseline, and situational factors at T53.

**Results:**

Complete IPV data were available for 301 women. At T0, 154 (51%) women reported lifetime history of physical IPV, of whom 62% gave a discrepant ‘never’ report in a subsequent round. Among 93 (31%) with lifetime history of sexual IPV at T0, 73% provided a subsequent discrepant report. 73% of women reported lifetime physical IPV, and 55% lifetime sexual IPV in at least one survey round. For both IPV outcomes, women were less likely to provide discrepant reports if they had recent IPV at baseline, poor mental health (T53) and poor communication with partner (T53). For physical IPV only, reduced discrepant reporting was also associated with baseline household-level financial hardship and more severe or extensive experience of IPV.

**Conclusions:**

A large proportion of women provided discrepant reports over the course of the study. Prevalence estimates of lifetime IPV from one-off cross-sectional surveys are likely to be underestimates, biased towards more recent and severe cases. To improve the stability of IPV measures, researchers should explicitly clarify the meaning of reference periods such as ‘ever’, consider using shorter reference periods (e.g. past-year), and avoid filter questions that use positive reports of lifetime IPV as a gateway to asking about more recent experiences.

*Trial registration***:** Maisha CRT01 registered at ClinicalTrials.gov #NCT02592252, registered retrospectively (13/08/2015).

**Supplementary Information:**

The online version contains supplementary material available at 10.1186/s12905-022-01697-y.

## Background

Violence against women (VAW) is a public health and human rights problem that is pervasive throughout the world. Recent estimates suggest that at least one quarter of women globally have experienced physical and/or sexual violence by a partner during their lifetime [1], with far reaching consequences for their mental and physical health [2–5]. The last decade has seen a growth in research to understand the extent, causes and consequences of intimate partner violence (IPV), and evaluate IPV prevention strategies [6, 7]. Surveys collecting self-reported data on violence experience and perpetration are key to these efforts.

The challenges of collecting accurate self-reports of women’s experience or men’s perpetration of IPV are considerable, with violence often underreported. Women may not disclose violence because of shame or stigma, fear for their physical safety, or even loyalty to the abuser[8]. Men may be even less likely to disclose perpetration[9–11], fearing social or legal repercussions, or simply choosing to give a ‘socially desirable’ response. Recall bias can also occur unrelated to the sensitive nature of the questions, where respondents forget events or forget the timing of when events occurred.

Many factors, including the wording and framing of questions, interviewer characteristics, interviewer training[12], protocols to ensure the safety of respondents, and mode of data collection have been shown to influence levels of disclosure of abuse[13].

Questions that ask about experience of behaviourally specific acts rather than using emotionally loaded terms like ‘abuse’, not only make measures of IPV more comparable across settings, but also lead to higher levels of disclosure[14]. Such behaviourally focused questions allow respondents to answer in the affirmative without having to identify as victims of ‘abuse’. Disclosure is also higher when women are given multiple opportunities to disclose, as when asked a series of questions on specific acts, rather than when a single general question on violence is used[8, 15]. This format aids recall as well as providing the respondent with several chances to psychologically ready themselves to disclose.

The careful selection and training of field-staff is also key to maximising disclosure[12, 16]. Women are more likely to disclose abuse if given a safe environment in which to do so, which is anyway an ethical imperative of research on VAW [16]. It is thus recommended practice for interviewers to be the same sex as the respondent, and trained on VAW, trauma, building rapport with respondents, and safety issues around the conduct of VAW research. Guidelines emphasise the importance of maintaining complete privacy during interviews, assuring respondent confidentiality, and having support and referral protocols in place for women in immediate danger or in need of follow-up support[17].

Evidence is mixed on how mode of survey delivery affects disclosure of violence or other sensitive or stigmatised behaviours or experiences. Several studies have shown disclosure of child sexual abuse (CSA) is higher when anonymous methods of data collection are used—such as asking respondents to anonymously indicate responses on a card which is then placed in a sealed envelope[18–20]. Similarly, sexual behaviour studies have shown that audio-computer assisted self-interviews (ACASI), which ensure anonymity, lead to higher levels of disclosure and more consistent and stable responses than face-to-face interviews[21, 22]. However, several studies have found ACASI to perform worse than face-to-face interviewing in eliciting women’s disclosure of forcibly being touched[23] and experiencing domestic violence[24, 25].

The WHO Multi-country Study on Women’s Health and Domestic Violence was the first study to use standardised measures, survey designs and interviewer training guidelines, to collect comparable data on VAW across diverse settings[26]. The standardised approach and measures used in this study have since been widely adopted and adapted; for example, in the Demographic and Health Surveys’ (DHS) standardised module on partner violence[27].

Though the WHO and DHS questions have been shown to have good construct validity and internal reliability in several settings[26, 28, 29], little is known about the stability of these measures over time. The test–retest reliability of a measure indicates the likelihood that a participant will provide the same response to the question(s) administered at two different time-points, where the time-interval between measurements is sufficiently short that the true response would not be expected to change. The little research that exists on the test–retest reliability of VAW measures more broadly relates predominantly to the USA and other VAW scales, most notably the Conflict Tactics Scale (CTS)[30, 31]. We know of just one study that has formally assessed the test–retest reliability of a VAW measure in the global South. Gibbs et al. (2019) used Cohen’s Kappa statistic to assess the stability of individuals’ responses to the WHO IPV questions among young people in South Africa[32]. They found only fair to moderate stability for ‘ever’ measures of physical IPV and sexual IPV, and lower stability for the ‘past year’ measures. Men’s reports consistently showed even lower stability than women’s reports, a pattern also observed in a study using a sexual violence measure in a US college sample[33]. However, this analysis was in a small sample of participants, which could have contributed to the low levels of stability.

Another indicator of the temporal stability of a measure is the extent to which those who report lifetime occurrence of behaviours or experiences at one point in time, subsequently report that these behaviours or experiences have never occurred. This method of considering logical inconsistencies in reporting—changes from ‘ever’ to ‘never’ reports—is one that can be usefully applied to research into the stability of lifetime measures of IPV. Such analyses can be conducted using data collected over longer time intervals than those required for a Cohen’s Kappa test–retest reliability study. Though there is a paucity of such analyses, longitudinal research into IPV reporting among women in Australia suggests considerable inconsistencies in women’s reporting of lifetime IPV experience over time[34, 35].

Little is known about which other factors besides gender are associated with the stability or otherwise of VAW measures. Gibbs et al. (2019) found completed secondary education to be associated with reduced odds of changed responses to questions on sexual IPV[32]. Similar observations have been made elsewhere in relation to stability of reporting of sexual behaviour/health measures[24, 36, 37]. Poor mental health has also been found to be associated with greater consistency in reporting of IPV[35].

We use data from the MAISHA CRT01 cluster randomised controlled trial, conducted in north-western Tanzania, and from an ongoing longitudinal study of control arm women to examine discrepancies in women’s reported experience of lifetime physical and sexual IPV over 53 months of follow-up [38]. We focus on changes that are logically inconsistent—changes from reports of ‘ever’ experiencing IPV to ‘never’ experiencing IPV—and examine the associations between these ‘discrepant’ reports and respondent baseline characteristics, the nature of their IPV experiences at baseline, and situational factors at the time of last follow-up.

## Methods

The MAISHA CRT01 trial evaluated the impact of a social empowerment IPV prevention intervention among women taking part in a group-based microfinance loan scheme in Mwanza city, Tanzania[39]. Informed consent was sought from each member of the microfinance group, and the group was enrolled into the trial if at least 70% of members consented. Sixty-six established microfinance loan groups (n = 1049 women) were enrolled, of which 33 groups (n = 544 women) were randomly allocated to the intervention (group-based gender training) and 33 (n = 505) to the control arm. At the end of the trial, women in the control arm were asked if they would be willing to take part in a follow-on study, which involved taking part in two further interviews. Informed consent was obtained from the women immediately prior to the follow-on interviews.

**Fig. 1:**
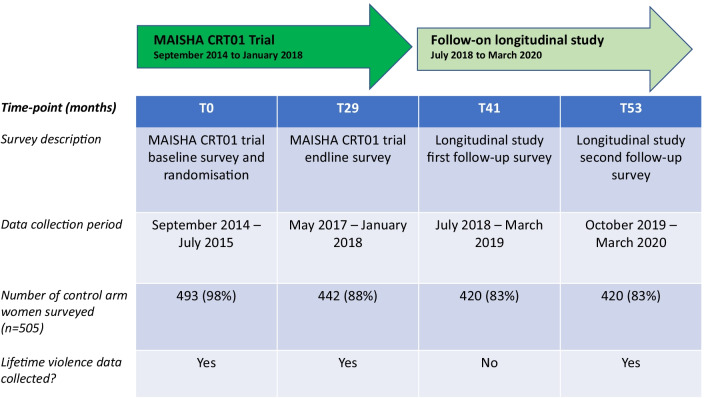
Data collection timeline

For the original trial, women were interviewed at trial baseline just prior to randomisation (T0) and again at trial follow-up, which was 29 months post-randomisation (T29). Women who consented to take part in the follow-on study, were interviewed at two further time points at yearly intervals, around 41 months post-randomisation (T41) and 53 months post-randomisation (T53) (See Fig. 1).

Interviews were conducted face to face, in private, by female interviewers trained in interviewing techniques, gender issues, violence and ethical issues related to research on IPV. The questionnaires were translated into Swahili and independently back translated into English. They included questions on the woman’s household, income, relationships, health, childhood, attitudes, and experiences of IPV. Responses were recorded directly onto tablet computers with validation checks to minimise missing or erroneous data. Data were uploaded daily to a secure database and checked by the data manager.

The study was conducted in accordance with WHO recommendations on researching violence against women[17]. The MAISHA trial received ethical approval from the Tanzanian National Health Research Ethics Committee of the National Institute for Medical Research (Ref: NIMR/HQ/R.8a/Vol.IX/1512), and the ethics committee of the London School of Hygiene and Tropical Medicine (Ref: 11,642). The longitudinal study also received ethical approval from the Tanzanian National Health Research Ethics Committee (Ref: NIMR/HQ/R.8a/Vol.IX/2475) and the London School of Hygiene and Tropical Medicine (Ref: 11,918 – 4).

### IPV outcomes

IPV questions were adapted from the WHO Violence Against Women instrument, with sexual violence questions extended to include situations where women are coerced into sexual acts not through physical force but out of fear of the consequences if they refuse. Respondents were asked whether they had ever experienced a series of specific acts, and if so whether they had experienced each in the past 12 months. Those reporting ‘yes’ to ever experiencing any of the physical acts were classified as having lifetime experience of physical IPV, those reporting ‘yes’ to ever experiencing any of the sexual acts were classified as having lifetime experience of sexual IPV, and those reporting at least one physical or sexual act were classified as having lifetime experience of physical and/or sexual IPV (Table 1).

**Table 1:** Questions used to construct the IPV outcomes

Lifetime physical IPV	Answers ‘yes’ to at least one of the following:
	Has your current partner or any other partner ever…
	Slapped you or thrown something at you that could hurt you?
	Pushed you or shoved you, or pulled your hair?
	Hit you with his fist or something else that could hurt you?
	Kicked you, dragged you or beaten you up?
	Choked or burnt you on purpose?
	Threatened to use or actually used a gun, knife or other weapon against you?
Lifetime sexual IPV	Answers ‘yes’ to at least one of the following:
	Has your current partner or any other partner ever forced you to have sexual intercourse by threatening you, holding you down or hurting you in some way?
	Have you ever had sexual intercourse when you did not want to because you were afraid that your partner would hurt you or someone you cared about if you refused?
	Have you ever had sexual intercourse when you did not want to because you were afraid that your partner would leave you or take another girlfriend if you refused?
Lifetime physical and/or sexual IPV	Experienced ‘physical IPV’ and/or ‘sexual IPV’ as defined above.

### Factors explored as potential correlates of discrepant reporting

- Details of the questions used to measure factors potentially associated with discrepant reporting of lifetime IPV are presented in Additional file 1. Briefly, three categories of potential correlates were considered:*Baseline demographics* included age (< 35 yrs; 35 +) and education (primary or below; above primary), both known correlates of IPV risk and factors which may affect how past events are interpreted and recalled. We also included past year financial hardship, a potential risk factor for ongoing violence—we hypothesised that those experiencing ongoing violence may be less likely to provide a discrepant ‘never’ report at follow-up time points than those for whom violence had ceased.
- *Features of IPV experience reported at baseline (timing, severity and extent)* included past year experience of the respective type of IPV (yes; no), reported fear of partner in the past year (yes; no), extent of lifetime experiences of physical and sexual IPV (one type; both types), lifetime experience of severe physical IPV (yes; no), and lifetime experience of emotional IPV (yes; no). We hypothesised that women with more recent experiences of IPV, and those who had experienced more severe or extensive forms of IPV would be more likely than others to persist in their reporting of lifetime IPV throughout the duration of the study.
- *Situational factors at T53* comprised partner change since T0 (same partner/left partner; new partner), poor mental health (no; yes), and good communication with partner (yes; no). We posited that women who had changed partners might no longer report IPV perpetrated by a past partner. Partner communication was selected as an indicator of relationship dynamics that could affect how a woman feels about her relationship and, in turn, her propensity to recall or report past negative experiences with her partner. A woman’s mental health at the time of follow-up could similarly affect her interpretation and recall of life-events.

### Statistical analysis

The analysis was restricted to those women providing data at all three time points at which lifetime experience of IPV was collected—T0, T29 and T53. Since the follow-on longitudinal study only asked IPV questions of women partnered in the past year, we also restricted the analysis to women who reported a past year partner at all three time-points. The main analyses were performed separately for physical and sexual IPV.

We first present lifetime prevalence of both types of IPV and a composite outcome of the two (physical and/or sexual), as reported by respondents at each of the three time points. We also present the cumulative total of women who report each type of lifetime IPV at least once during the course of the study.

The analysis of discrepancies in reporting of IPV pertains only to those reporting lifetime experience of each type of IPV at T0. For women reporting lifetime experience of each type of IPV at T0, we calculated the percentage who changed to reporting ‘never’ experiencing that type of IPV in a subsequent round (T29 and/or T53). These subsequent ‘never’ reports were classed as ‘discrepant’ reports, as they are logically inconsistent with the woman’s first report.

Among the subset of women reporting lifetime experience of each type of IPV at T0, we explored factors associated with discrepant reporting in any subsequent study round, using cross-tabulations and logistic regression with robust standard errors to account for the clustered nature of the data. Two categories of associated factors were considered: baseline demographics; and features of IPV experience reported at baseline (timing, severity and extent). We also explored the association between situational factors at T53 (partner change since T0, quality of communication with partner, respondent mental health) and discrepant reporting between T0 and T53 only. No adjustment has been made for multiplicity due to the exploratory nature of this analysis[40].

We also performed a sensitivity analysis in order to assess whether observed associations reflected factors associated with continued *experience* of IPV rather than with discrepant/continued *reporting* of IPV. First, we used logistic regression to explore the association between the baseline indicators (respondent characteristics and features of IPV experience) and discrepant reporting between T0 and T53 only (excluding T29). We then repeated this analysis of factors associated with discrepant reporting between T0 and T53, excluding respondents with past year experience of the respective type of IPV at T53—i.e. restricted to women with lifetime but not persistent experience of IPV.

All analyses were performed using Stata 17.

## Results

### Response rates and participant baseline characteristics

Among the 505 women in the control arm of the MAISHA trial, 493 (98%) completed a baseline interview (T0) and 395 (78%) were interviewed at all three time points at which ‘ever’ IPV data were collected (T0, T29 and T53). Of these, 301 (76%) reported a past year partner at all three time points and are thus included in this analysis.

Women included in the analysis were broadly similar to women in the overall baseline sample with respect to a range of demographic characteristics, though were more likely than women in the overall sample to be currently married at baseline. The baseline (T0) mean age of women who reported a past year partner at all three time points was 38.8 years (range 19–66) (Table 2). Most women were married or living as married at baseline (86%), and almost all (98%) had had children. Only 27% had attended secondary education or higher, and 42% reported that their household had experienced financial hardship (difficulty covering basic household expenses) in the year preceding the survey.

**Table 2:** Baseline (T0) characteristics of respondents

	Entire baseline sample (N = 493) n (%)	Women included in the analysis (N = 301)^a^ n(%)
Age (yrs) *Mean (sd) [range]*	40.2 (9.5) [19–70]	38.8 (9.0) [19–66]
Marital status		
*Married*	360 (73%)	258 (86%)
*Divorced/separated*	74 (15%)	28 (9%)
*Widowed*	47 (10%)	8 (3%)
*Never married*	12 (2%)	7 (2%)
Highest level of education completed		
*None/incomplete primary*	61 (12%)	30 (10%)
*Completed primary*	298 (60%)	190 (63%)
*Attended secondary/higher*	134 (27%)	81 (27%)
Household experienced financial hardship in past year	207 (42%)	127 (42%)
Number of children respondent has given birth to		
*None*	10 (2%)	7 (2%)
*1–2*	96 (19%)	57 (19%)
*3–4*	196 (40%)	123 (41%)
*5* +	191 (39%)	114 (38%)

^a^ Women who reported having a partner in the 12 months prior to interview and who provided data on ever IPV at T0, T29 and T53

### Prevalence of ‘ever’ IPV measures at T0, T29 and T53

The prevalence of women reporting lifetime IPV declined over the course of the study, particularly between T29 and T53 (Table 3). The decline was more marked for physical IPV (from 51% at T0 to 40% at T53, a 22% reduction) than sexual IPV (from 31% at T0 to 26% at T53, a 16% reduction).

**Table 3:** Prevalence of women reporting ever having experienced IPV in different rounds of the study (n = 301)

Ever experienced:	T0	T29	T53	Lifetime IPV reported in any of T0, T29, or T53
Physical IPV	154 (51%)	147 (49%)	120 (40%)	220 (73%)
Sexual IPV	93 (31%)	103 (34%)	78 (26%)	166 (55%)
Physical and/or sexual IPV	177 (59%)	173 (57%)	144 (48%)	239 (79%)

Over the course of the study, 73% of women reported lifetime experience of physical IPV in at least one interview. The corresponding figures for sexual IPV and physical and/or sexual IPV were 55% and 79% respectively (Table 3).

### Percentage of women providing ‘discrepant’ reports (change from ‘ever’ to ‘never’ reports of IPV)

Among women reporting lifetime experience of each type of IPV at T0, levels of discrepant reporting were high, increasing throughout the study (Table 4). For those reporting lifetime physical IPV at T0, 62% went on to report never having experienced physical IPV in at least one subsequent interview. The respective figure for sexual IPV was even higher at 73%, and for physical and/or sexual IPV comparatively lower at 55%. Discrepant reporting was markedly higher at T53, as opposed to T29 for all measures.

**Table 4:** Percentage of women reporting ever IPV at T0 who report never having experienced IPV in subsequent interviews

Ever experienced:	N reporting IPV at T0	T29–report ‘Never’	T53–report ‘Never’	T29 and/or T53–report ‘Never’
Physical IPV	154	59 (38%)	75 (49%)	96 (62%)
Sexual IPV	93	40 (43%)	54 (58%)	68 (73%)
Physical and/or sexual IPV	177	49 (28%)	76 (43%)	97 (55%)

### Factors associated with discrepant reporting

#### Baseline characteristics

Among women who reported lifetime experience of IPV at T0, neither the respondent’s age nor education were associated with odds of giving discrepant ‘never’ reports at T29 and/or T53. Those who reported baseline household level financial hardship were less likely to provide discrepant reports for physical IPV than those without financial hardship (OR = 0.48, 95% CI 0.24–0.98) (Table 5).

**Table 5:** Baseline factors associated with discrepancies in IPV reporting between T0 and any subsequent round (among women reporting ever having experienced each type of IPV at T0)

Baseline indicator	Physical IPV (n = 154)		Sexual IPV (n = 93)	
	Discrepant reporting of physical IPV	OR (95% CI)	Discrepant reporting of sexual IPV	OR (95% CI)
Age				
*Under 35*	30/53 (57%)	-	24/34 (71%)	-
*35* +	66/101 (65%)	1.45 (0.59–3.57)	44/59 (75%)	1.22 (0.44–3.42)
	*p* = *0.287*	*p* = *0.424*	*p* = *0.676*	*p* = *0.702*
Education				
*Primary or below*	73/115 (63%)	-	49/65 (75%)	-
*Above primary*	23/39 (59%)	0.83 (0.47–1.46)	19/28 (68%)	0.69 (0.28–1.67)
	*p* = *0.616*	*p* = *0.514*	*p* = *0.453*	*p* = *0.410*
Household-level financial hardship in past year				
*No*	57/81 (70%)	-	30/39 (77%)	-
*Yes*	39/73 (53%)	0.48 (0.24–0.98)	38/54 (70%)	0.71 (0.30–1.71)
	*p* = *0.030*	*p* = *0.045*	*p* = *0.482*	*p* = *0.446*
Past year experience of this type of IPV				
*No*	63/92 (68%)	-	40/48 (83%)	-
*Yes*	33/62 (53%)	0.52 (0.26–1.05)	28/45 (62%)	0.33 (0.12–0.89)
	*p* = *0.055*	*p* = *0.069*	*p* = *0.022*	*p* = *0.029*
Fear of partner in past year				
*Never*	78/105 (74%)	-	43/55 (78%)	-
*A few times*	11/26 (42%)	0.25 (0.10–0.63)	13/18 (72%)	0.73 (0.24–2.15)
*Many/most/all of time*	7/23 (30%)	0.15 (0.07–0.34)	12/20 (60%)	0.42 (0.13–1.34)
	*p* < *0.001*	*p* < *0.001*	*p* = *0.290*	*p* = *0.304*
Ever experience of one or more types of IPV (physical and/or sexual)				
*One*	60/84 (71%)	-	17/23 (74%)	-
*Both*	36/70 (51%)	0.42 (0.20–0.90)	51/70 (73%)	0.95 (0.28–3.15)
	*p* = *0.033*	*p* = *0.039*	*p* = *0.921*	*p* = *0.930*
Ever severe physical IPV				
*No*	33/46 (72%)	-	-	-
*Yes*	63/108 (58%)	0.55 (0.26–1.16)	-	-
	*p* = *0.116*	*p* = *0.115*	*-*	*-*
Ever emotional IPV				
*No*	20/22 (91%)	-	12/15 (80%)	-
*Yes*	76/132 (58%)	0.14 (0.03–0.56)	56/78 (72%)	0.64 (0.19–2.11)
	*p* = *0.003*	*p* = *0.006*	*p* = *0.512*	*p* = *0.460*

#### Baseline IPV experience

While all women included in this analysis reported lifetime experience of IPV at baseline, the timing, extent and severity of that IPV varied across the sample. Many of these aspects of women’s IPV experience at baseline were associated with odds of discrepant reporting in a later round of the study.

For both physical IPV and sexual IPV, women who had *past year* experience of the respective type of IPV at baseline were less likely than those without past year experience to provide discrepant reports in a subsequent round (physical IPV, OR = 0.52, 95% CI 0.26–1.05; sexual IPV, OR = 0.33, 95% CI 0.12–0.89). Increasing frequency with which women reported fearing their partner in the past year was also associated with progressively decreasing odds of discrepant ‘never’ reports, an association that was only statistically significant in relation to physical IPV (Table 5).

Other aspects of lifetime experience of IPV were related to propensity for discrepant reporting in relation to physical IPV but not sexual IPV. Those who had lifetime experience of both physical and sexual IPV were less likely to give discrepant reports for physical IPV than those who had experienced just one kind of IPV (OR = 0.42, 95% CI 0.20–0.90). Women who had lifetime experience of severe physical IPV were also less likely to give discrepant reports (not statistically significant), as were those who had lifetime experience of emotional IPV alongside physical IPV (OR = 0.14, 95% CI 0.03–0.56) (Table 5).

#### Situational factors at T53

There was weak evidence of an association between partner change and odds of discrepant reporting for the physical IPV outcome at T53. Women who changed partner between T0 and T53 were more likely to provide discrepant reports than women who remained with their partner throughout the study or were recently separated before T53 (not statistically significant) (Table 6).

**Table 6:** T53 situational factors associated with discrepancies in IPV reporting between T0 and T53 (among women reporting ever having experienced each type of IPV at T0)

T53 situational factors	Physical IPV (n = 154)		Sexual IPV (n = 93)	
	Discrepant reporting of physical IPV	OR (95% CI)	Discrepant reporting of sexual IPV	OR (95% CI)
Changed partner since baseline				
*Same partner/left partner*	58/125 (46%)	-	39/68 (57%)	-
*New partner*	17/29 (59%)	1.64 (0.60–4.43)	15/25 (60%)	1.12 (0.41–3.01)
	*p* = *0.236*	*p* = *0.332*	*p* = *0.819*	*p* = *0.829*
Poor mental health				
*No*	65/120 (54%)	-	45/70 (64%)	-
*Yes*	10/34 (29%)	0.35 (0.12–1.00)	9/23 (39%)	0.36 (0.13–0.96)
	*p* = *0.011*	*p* = *0.051*	*p* = *0.034*	*p* = *0.041*
Communicates well with partner				
*No*	23/62 (37%)	-	12/34 (35%)	-
*Yes*	52/92 (57%)	2.20 (1.04–4.68)	42/59 (71%)	4.53 (2.08–9.85)
	*p* = *0.018*	*p* = *0.040*	*p* = *0.001*	*p* < *0.001*

Women with poor mental health at T53 were less likely to give discrepant reports (at T53) for physical IPV and sexual IPV than women without poor mental health (physical IPV, OR = 0.35, 95% CI 0.12–1.00; sexual IPV, OR = 0.36, 95% CI 0.13–0.96). Women who reported good communication with their partner at T53 were more likely to give discrepant reports than women in relationships characterised by less good communication (physical IPV, OR = 2.20, 95% CI 1.04–4.68; sexual IPV, OR = 4.53, 95% CI 2.08–9.85) (Table 6).

### Results of sensitivity analyses

The results of the associated factors analysis were similar when looking at discrepant reporting between T0 and T53 only (Additional file 2).

When women with past year experience of each type of IPV at T53 were excluded from the respective analyses–i.e. including only women for whom experience of IPV had not persisted over the duration of the study—overall levels of discrepant reporting were higher. Though some associations weakened slightly, patterns of association remained similar (Additional file 3).

## Discussion

This analysis yields findings that have important implications for how we conduct and interpret IPV research. At the individual-level, we show high levels of discrepant reporting over time for measures of lifetime IPV experience, with discrepant reporting defined as reporting lifetime experience of IPV at T0, but reporting no lifetime history of IPV at T29 and/or T53. We identify several factors associated with discrepant reporting over time. Most notably, these relate to the recency, severity and extent of the IPV experienced. Women with more recent experience of IPV, and experience of multiple types or severe acts of IPV at baseline were less likely to provide discrepant reports at subsequent time points than those with less recent, less extensive and less severe IPV. Situational factors at the subsequent time point, such as women’s poor mental health and poor communication with her partner, were also associated with reduced discrepant reporting. Over the course of the study, the cumulative percentage of women reporting lifetime experience of IPV in at least one survey round (73% for physical IPV and 55% for sexual IPV) was very high and far exceeded that reported in any single round including the final round (T53). This suggests that the prevalence of life-time violence is far higher than we normally assume.

The finding on high levels of discrepant reporting builds on evidence from the only other study we know of that has explored the stability of WHO based IPV measures at the individual level. In two surveys spaced 2 weeks apart, Gibbs et al. (2019) found only fair to moderate stability for the measure of ‘ever physical IPV’ (k0.58) and ‘ever sexual IPV’ (k0.56) among women [32]. While agreement over time was higher than in our analysis (81.8% for ever physical and 78.6% for ever sexual), it is important to note that the Gibbs study assessed reporting over a much shorter time period (2 weeks) and assessed all discordant reports (‘ever’ to ‘never’ as well as ‘never’ to ‘ever’). They found changes from ‘ever’ to ‘never’ to be more common than those from ‘never’ to ‘ever’ for physical IPV (13.6% versus 5.4%), though changes in both directions were equally common for sexual IPV (9.8% versus 11.6%) (personal communication)[32].

Other longitudinal research into IPV reporting has also found high levels of inconsistencies in reporting using a single-item measure. Among Australian women reporting lifetime IPV experience at least once over a 20-year study period (comprising 6 survey rounds), 54% were inconsistent in their reporting throughout the study[34]. Likewise, a longitudinal survey of 18–23 year-old Australian women, found that a third of women who reported lifetime IPV in one survey round, subsequently reported no history of IPV 12 months later[35]. Similar inconsistencies have been found in relation to other experiences of abuse and trauma. In two surveys conducted 4–6 weeks apart among a sample of Dutch adults, 35% of those who reported extra-familial child sexual abuse (CSA) during the first survey, did not report CSA in the second survey[41]. Among a community-based cohort in Switzerland, 40% of participants who reported a potentially traumatic event in an initial survey, did not report the event when surveyed again 6 years later[42]. In a study of the stability of teacher’s self-reports of perpetration of physical violence against students before and after a one-day violence prevention training in Cote d’Ivoire, the percentage of teachers reporting lifetime perpetration of any violent act fell from 73% (immediately prior to training) to 47% (immediately post training)[43].

Research in other fields also helps to put our findings into context. Low stability of self-reported lifetime ever/never measures has been extensively documented in relation to other sensitive health topics, such as alcohol and drug use among adolescents [44–47] where the switch from reports of ‘ever use’ to ‘never use’ is referred to as ‘recanting’. Fendrich and Rosenbaum (2003), for example, found rates of recanting for lifetime reports of alcohol and cocaine use among adolescents to be 45% and 81% respectively over 6 years of follow-up[44]. Recanting has also been observed in relation to self-reports of sexual behaviour. Among young men in the United States of America, Dariotis et al. found that 94–98% who reported ever having had a sexually transmitted infection recanted their reports in a later wave of the 9 year study[48]. A study of South African adolescents, surveyed at five 6-monthly intervals, found that nearly 40% of respondents who reported being sexually active in an early wave of data collection reported being a virgin in a later wave[36].

We have several hypotheses to explain the high levels of discrepant reporting that we observed, some of which relate to respondents’ interpretations of the reference period for IPV questions. Though questions on lifetime IPV ask women whether they have ‘ever’ experienced specific acts, it is possible that women in later study rounds interpret this to relate to the period since they were last interviewed. Anecdotal reports from fieldworkers suggest that women sometimes assumed they were being asked about IPV in the past year only since this had been the reference period for preceding questions on relationship characteristics and dynamics. Since this analysis is being conducted retrospectively, we are unable to explore the impact that changes to wording, explanation or placing of questions in the questionnaire might have on the performance of the measures. It is also possible that respondents were affected by respondent fatigue, a phenomenon noted by researchers analysing longitudinal data on a range of topics [49–51]. They may be unwilling to respond to the same questions asked repeatedly, especially once they learn that answering in the affirmative leads to a set of further sub-questions. Responses may also be influenced by a woman’s feelings or mood at the time of the interview–there may be times when she feels able to discuss past trauma and other times when she does not.

In addition to demonstrating high levels of discrepant reporting for lifetime IPV measures, our analysis also provides important insights into respondent-level factors which may be associated with discrepant reporting of IPV. As with Gibbs et al.’s (2019) analysis of the test–retest stability of the WHO measures[32], we found no association between the respondent’s age and odds of discrepant reporting. We also, in contrast with Gibbs’ findings, found no association between secondary education and reduced odds of discrepant reporting. We did, however, observe an association between past year experience of financial hardship at baseline and reduced odds of discrepant reporting. Financial hardship (and other indicators of socio-economic status) are strong risk factors for IPV[52, 53]. It is plausible that women living in households that experienced financial hardship were more likely to experience ongoing IPV, and therefore to persist in reporting lifetime experience of IPV at follow-up time-points.

Importantly, we found strong associations between discrepant reporting of lifetime IPV and the timing, severity and extent of the IPV initially reported. These findings are in keeping with the results of an analysis looking at retrospective self-reports of CSA, in which respondents with less severe abuse were more likely to provide inconsistent reports than those with more severe abuse[41]. Associations have also been found between the temporal stability of other self-reported health measures and greater severity/dose of the initial reports, for example in relation to alcohol dependence[54], illicit drug use[55] and cigarette smoking[56]. It is plausible that more serious events or extreme behaviours are more readily recalled and deemed worthy of report than those that had less of an impact on an individual’s life. Our observation that women who also experienced emotional IPV were much less likely to provide discrepant reports about lifetime experience of physical IPV than those who experienced physical IPV alone, is a reminder of the deep and lasting impacts that emotional IPV can have on women’s health and well-being[57].

We also found situational factors at T53 to be associated with discrepant reports. There was suggestive evidence that women who had a new partner since T0 were more likely to change from ‘ever’ to ‘never’ reports than women who had remained with the same partner throughout the study. It is plausible that women who have changed partner view experiences with a past partner as less relevant to their current lives, or as events from which they wish to ‘move on’. Women in relationships characterised by good communication at T53 were also more likely to give discrepant reports at T53, potentially because they reinterpret past events in light of current feelings towards their partner. Anecdotal reports from fieldworkers also suggest the strong role of ‘forgive and forget’, with women not wanting to reopen old wounds if a relationship has since improved. Conversely, women with poor mental health were less likely to give discrepant reports. It is possible that these women were more likely to be in persistently violent relationships, and may also reflect the continued feeling of relevance that past events have for their lives. Our findings concord with other analyses of inconsistencies in reporting of IPV[35], and with those of a study looking at inconsistent reporting of self-harm, in which inconsistent reporters were less likely than consistent reporters to have depression[58].

This study has several limitations. The first of these is respondent attrition, to which longitudinal research is particularly prone. Nevertheless, we obtained complete data for 78% of women in our study (before further restricting this analysis to women partnered at all three time-points), a good response rate for a longitudinal study. While it is possible that those remaining in the study differ in important ways from those lost to follow-up, an analysis of baseline data suggests this was not a major source of bias in this study.

Since this study was not set up as a test–retest reliability study, we have only been able to assess reporting changes from ‘ever’ to ‘never’, similar to studies reporting on ‘recanting’, rather than all possible inconsistencies in reporting. However, while this precludes an assessment of the measure’s test–retest reliability via Cohen’s Kappa (indicative of its overall ‘performance’), our findings are arguably more useful for assessing inconsistencies in reporting of lifetime IPV experience over time-frames relevant to longitudinal IPV research. We have also only explored discrepancies in women’s self-reported experience, and our findings cannot be generalised to men’s reports of perpetration, to women in different socio-economic, cultural or geographical contexts, or to men experiencing IPV within heterosexual or same-sex relationships. This analysis was also not able to examine all factors that might affect the stability of the IPV measures. For example, too few women had been consistently interviewed by the same interviewer across rounds to be able to examine whether change in interviewer influenced changes in reporting. Changes in interviewer could plausibly affect discrepant reporting in several ways; a woman might believe it unnecessary to tell the same interviewer the same experience twice (hence switching to ‘never’ reports after reporting IPV in an earlier round), or she might choose not to relive the same reporting experience twice to two different interviewers. Furthermore, disclosure may depend on the rapport she feels with any given interviewer. We could also not assess the impact of different forms of questionnaire administration, for instance ACASI, or using cards, as compared to face-to-face interviews.

This study also has many strengths, not least that it addresses an important methodological issue regarding measurement of IPV. The WHO instrument is widely used, and yet there is a dearth of evidence on the stability of the measures over time. The analysis has been made possible by the availability of longitudinal data collected over multiple time-points, still a relative rarity in IPV research to date. We have not only highlighted high levels of discrepant reporting for lifetime measures of IPV, but also the extent to which cross-sectional studies (that ask IPV questions at a single timepoint) may underestimate ‘true’ prevalence of lifetime IPV experience. 53% of women reported lifetime experience of physical IPV at T0, but 74% reported lifetime physical IPV in at least one round of the study. Furthermore, we have explored associations between discrepant reporting and a range of factors relating to respondent characteristics and experiences at different reporting time-points. Our sensitivity analyses have allowed us to confirm that the associations we observe are not just artefact of those same factors being risk factors for continued experience or cessation of IPV, but also reflect differences in reporting behaviour. Patterns of association persist even once women with past year IPV at T53 are excluded from the analysis.

Our findings have implications both for how we interpret current estimates of lifetime prevalence of violence and how we conduct IPV research in future. First, given the high rates at which women switch from ‘ever’ to ‘never’ reports over relatively short time-frames, it is reasonable to assume that the ‘true’ percentage of women who have ever experienced IPV is considerably higher than that reported at any one point in time–in order to gain true estimates of prevalence or effectively screen for IPV, it may be necessary to ask women about IPV on several separate occasions. Second, given that women with more recent, severe or extensive experiences of IPV are less likely to switch to never reports, prevalence estimates of lifetime IPV can be reinterpreted as biased towards the more recent and severe cases of IPV. Third, in order to improve on the stability of measures of IPV, methodological revisions may be necessary in IPV research. Lifetime measures may be best collected at the start of a study, with shorter reference periods used in subsequent study rounds. Research is also needed into whether wording changes might decrease levels of discrepant reporting–for example, where lifetime measures are repeated, explicitly clarifying that the term ‘ever’ means ‘ever in your life’. Furthermore, the reference period of questions must be carefully stated and remain as consistent as possible within questionnaires, with clarifying introductory sentences used to alert respondents when the time-frame of interest has changed for a specific set of questions. Lastly, it is common practice to use questions on lifetime IPV experience as a gateway to questions on more recent experience. Given the extent of under-reporting evident for lifetime experiences, the use of such filter questions should be reconsidered due to their potential to lead to a knock-on underestimation of more recent violence.

Finally, while our analysis has provided important insights into the stability of measures of lifetime experience of physical and sexual IPV, important evidence gaps remain. Future research is needed into the extent and correlates of discrepant reporting of emotional IPV, feeding into broader ongoing debates around the conceptualisation and measurement of emotional abuse[59], and to explore the stability of men’s reports of IPV perpetration.

## Supplementary Information

**Additional file 1.** Indicators explored as potential correlates of discrepant reporting of lifetime IPV. Details of question items used to construct indicators for analysis of factors associated with discrepant reporting of lifetime IPV

**Additional file 2. ** Factors associated with discrepancies in IPV reporting between T0 and T53 (among women reporting ever having experienced each type of IPV at T0). Odds ratios (and 95% confidence intervals) of associations between baseline/T53 situational factors and discrepancies in IPV reporting between T0 and T53, among women reporting ever having experienced each type of IPV at T0.

**Additional file 3.** Factors associated with discrepancies in IPV reporting between T0 and T53, excluding women with past year experience of the respective type of IPV at T53 (among women reporting ever having experienced each type of IPV at T0). Odds ratios (and 95% confidence intervals) of associations between baseline/T53 situational factors and discrepancies in IPV reporting between T0 and T53, excluding women with past year experience of the respective type of IPV at T53 (among women reporting ever having experienced each type of IPV at T0)

## Data Availability

Data underlying the primary trial analysis of the MAISHA CRT01 data is available upon request (with restricted access) from Data Compass, the London School of Hygiene digital data repository (https://doi.org/10.17037/DATA.00001775). The longitudinal study is still ongoing, and datasets are therefore not publicly available at this point. At the conclusion of our main study analyses, data will be made available upon request and in accordance with NIMR, LMU and LSHTM regulations.

## Abbreviation

ACASI: Audio-computer assisted self-interview
CI: Confidence interval
CRT: Cluster randomised trial
CSA: Child sexual abuse
CTS: Conflict tactics scale
DHS: Demographic and health surveys
IPV: Intimate partner violence
OR: Odds ratio
VAW: Violence against women
WHO: World health organization
