# Supplementary material for: Longitudinal inconsistencies in women’s self-reports of lifetime experience of physical and sexual IPV: evidence from the MAISHA trial and follow-on study in North-western Tanzania
Source: BMC Womens Health. 2022 Apr 15;22:120. doi: 10.1186/s12905-022-01697-y (PMC9013096; doi:10.1186/s12905-022-01697-y)
Supplement: Supplementary file 1 — Additional file 1. Indicators explored as potential correlates of discrepant reporting of lifetime IPV. Details of question items used to construct indicators for analysis of factors associated with discrepant reporting of lifetime IPV [file 12905_2022_1697_MOESM1_ESM.docx]

Additional File 1: Indicators explored as potential correlates of discrepant reporting of lifetime IPV

| **INDICATOR** | **HOW MEASURED** |
| --- | --- |
| **Baseline (T0) demographics** |  |
| Age | ***Binary: <35 years; 35+*** |
| Highest level of education completed | ***Highest completed level of education: Primary or below; Above primary*** |
| Household-level financial hardship in past year | ***Binary: Yes; No***  Respondent was asked a series of questions:  During the last 12 months…   1. how many times were you very worried/stressed about your general financial situation? 2. have you had trouble buying food or other necessities for your family 3. have you had to borrow money to pay rent or other bills? 4. did any of your family members need to see a doctor but could not because you did not have enough money? 5. did your children miss days of school because you did not have money for school fees, uniform or supplies? 6. Have you or any of your children gone a whole day without eating anything because there was not enough food?   (Response options: Never; Once; Few times; Many times)  Respondents were coded as having experienced household-level hardship in the past year if they answered:   - ‘A few times’ or ‘Many times’ to (a)   *and*   - either:   - ‘A few times’ or ‘Many times’ to any of (b)-(f)   *or*   - - ‘Once’ to at least two of (b)-(f) |
| Current marital status | ***Binary: Not married/living as married; Married/living as married*** |
| **Baseline (T0) experiences of IPV** |  |
| Past year physical IPV | ***Binary: Yes; No***  Coded as ‘Yes’ if she reports that a partner has done at least one of the following things to her in the past year:   - Slapped her or thrown something at her that could hurt her - Pushed her or shoved her or pulled her hair - Hit her with his fist or something else that could hurt her - Kicked her, dragged her or beat her up - Choked or burnt her on purpose - Threatened to use or actually used a gun, knife or other weapon against her |
| Past year sexual IPV | ***Binary: Yes; No***  Coded as ‘Yes’ if she reports that any of the following have happened to her in the past year:   - A husband/partner forced her to have sexual intercourse by threatening her, holding her down or hurting her in some way - She had sexual intercourse when she did not want to because she was afraid that her partner would hurt her or someone she cared about if she refused - She had sexual intercourse when she did not want to because she was afraid that her partner would leave her or take another girlfriend if she refused |
| Past year physical and/or sexual IPV | ***Binary: Yes; No***  Coded as ‘yes’ if reports either physical or sexual IPV in the past year (as defined above) |
| Fear of partner in past year | ***Categorical: Never; A few times; Many times/most/all of the time***  Respondents asked: “Thinking back over the past 12 months, would you say that you have been very afraid of your partner never, a few times, many times or most of the time?” |
| Ever experienced both types of IPV | One type (physical *or* sexual); Both (physical *and* sexual) (As defined in Table 1) |
| Ever experienced severe physical IPV | ***Binary: Yes; No***  Coded as ‘Yes’ if she reports that a partner has ever done at least one of the following things to her:   - Hit her with his fist or something else that could hurt her - Kicked her, dragged her or beat her up - Choked or burnt her on purpose - Threatened to use or actually used a gun, knife or other weapon against her   *Or*  that a partner has done at least one of the following things to her many times:   - Slapped her or thrown something at her that could hurt her - Pushed her or shoved her or pulled her hair |
| Ever experienced emotional IPV | ***Binary: Yes; No***  Coded as ‘Yes’ if she reports that a partner has ever done at least one of the following things to her:   - Insulted her or made her feel bad about herself - Belittle or humiliated her in front of other people - Done things to scare or intimidate her on purpose (e.g. by the way he looked at her, by yelling and smashing things) - Verbally threatened to hurt her or someone she cares about |
| **Situational factors at T53** |  |
| Changed partner since baseline | Categorical: No change; New partner; Left partner (currently single) |
| Poor mental health | Binary: Poor mental health; No mental health condition  Coded as ‘poor mental health’ if scored 8 or more on the SRQ-20. This asks respondents “During the last 4 weeks, have you been bothered by any of the following problems:   - Do you often have headaches? - Is your appetite poor? - Do you sleep badly? Like difficulties falling asleep, waking up in the middle of the night more than 3 times, or waking up early in the morning and not getting back to sleep. - Are you easily frightened? - Do your hands shake? - Do you feel nervous, tense or worried? - Is your digestion poor? Like you are often constipated, feel nauseous, or you don’t have an appetite. - Do you have trouble thinking clearly? - Do you feel unhappy? - Do you cry more than usual? Like everyday or more than once per day, because of problems? - Do you find it difficult to enjoy your daily activities? - Do you find it difficult to make decisions? - Is your daily work suffering? - Are you unable to play a useful part in life? - Have you lost interest in things? - Do you feel that you are a worthless person? - Has the thought of ending your life been on your mind? - Do you have uncomfortable feelings in your stomach? - Are you easily tired? |
| Good communication with partner | ***Binary: Yes; No***  Respondent asked: “During the last 12 months, did you and your partner discuss the following topics together:   - Things that happened to you during the day? - Things that happened to him in the day? - Your worries or feelings? - His worries or feelings?”   (Response options: Never; Once; Few times; Many times)  Coded as ‘Yes’ if discussed all of these at least ‘a few times’. Otherwise coded as ‘No’. |
