# Supplementary material for: Longitudinal inconsistencies in women’s self-reports of lifetime experience of physical and sexual IPV: evidence from the MAISHA trial and follow-on study in North-western Tanzania
Source: BMC Womens Health. 2022 Apr 15;22:120. doi: 10.1186/s12905-022-01697-y (PMC9013096; doi:10.1186/s12905-022-01697-y)
Supplement: Supplementary file 2 — Additional file 2. Factors associated with discrepancies in IPV reporting between T0 and T53 (among women reporting ever having experienced each type of IPV at T0). Odds ratios (and 95% confidence intervals) of associations between baseline/T53 situational factors and discrepancies in IPV reporting between T0 and T53, among women reporting ever having experienced each type of IPV at T0. [file 12905_2022_1697_MOESM2_ESM.docx]

Additional File 2: Factors associated with discrepancies in IPV reporting between T0 and T53 (among women reporting ever having experienced each type of IPV at T0)

| Baseline indicator | Physical  (n=154) | OR (95%CI) | Sexual  (n=93) | OR (95%CI) |
| --- | --- | --- | --- | --- |
| ***Discrepant reporting at T53*** | ***75/154 (49%)*** |  | ***54/93 (58%)*** |  |
|  |  |  |  |  |
| Age |  |  |  |  |
| *Under 35* | 25/53 (47%) | - | 23/34 (68%) | - |
| *35+* | 50/101 (50%) | 1.10 (0.47 – 2.54) | 31/59 (53%) | 0.53 (0.23 – 1.22) |
|  | *p=0.783* | *p=0.827* | *p=0.155* | *p=0.136* |
| Education |  |  |  |  |
| *Primary or below* | 57/115 (50%) | - | 37/65 (57%) | - |
| *Above primary* | 18/39 (46%) | 0.87 (0.49 – 1.54) | 17/28 (61%) | 1.17 (0.48 – 2.83) |
|  | *p=0.713* | *p=0.637* | *p=0.734* | *p=0.729* |
| Household-level financial hardship in past year |  |  |  |  |
| *No* | 45/81 (56%) | - | 26/39 (67%) | - |
| *Yes* | 30/73 (41%) | 0.56 (0.14 – 1.27) | 28/54 (52%) | 0.54 (0.25 – 1.16) |
|  | *p=0.073* | *p=0.124* | *p=0.153* | *p=0.114* |
| Past year experience of this type IPV |  |  |  |  |
| *No* | 49/92 (53%) | - | 34/48 (71%) | - |
| *Yes* | 26/62 (42%) | 0.63 (0.29 – 1.38) | 20/45 (44%) | 0.33 (0.15 – 0.74) |
|  | *p=0.168* | *p=0.249* | *p=0.010* | *p=0.007* |
| Fear of partner in past year |  |  |  |  |
| *Never* | 60/105 (57%) | - | 37/55 (67%) | - |
| *A few times* | 9/26 (35%) | 0.40 (0.16 – 1.00) | 10/18 (56%) | 0.61 (0.20 – 1.87) |
| *Many/most/all of time* | 6/23 (26%) | 0.26 (0.10 – 0.68) | 7/20 (35%) | 0.26 (0.10 – 0.72) |
|  | *p=0.008* | *p=0.005* | *p=0.042* | *p=0.032* |
| Ever experience of one or more types of IPV (physical and/or sexual) |  |  |  |  |
| *One* | 49/84 (58%) | - | 16/23 (70%) | - |
| *Both* | 26/70 (37%) | 0.42 (0.23 – 0.76) | 38/70 (54%) | 0.52 (0.19 – 1.44) |
|  | *p=0.009* | *p=0.004* | *p=0.198* | *p=0.208* |
| Ever severe physical IPV |  |  |  |  |
| *No* | 25/46 (54%) | - | - | - |
| *Yes* | 50/108 (46%) | 0.72 (0.35 – 1.48) | - | - |
|  | *p=0.360* | *p=0.378* | *-* | *-* |
| Ever emotional IPV |  |  |  |  |
| *No* | 15/22 (68%) | - | 12/15 (80%) | - |
| *Yes* | 60/132 (45%) | 0.39 (0.14 – 1.10) | 42/78 (54%) | 0.29 (0.10 – 0.82) |
|  | *p=0.048* | *p=0.076* | *p=0.060* | *p=0.020* |
| **T53 situational variables** |  |  |  |  |
| Changed partner since baseline |  |  |  |  |
| *No change/ left partner* | 58/125 (46%) | - | 38/63 (60%) | - |
| *New partner* | 17/29 (59%) | 1.64 (0.60 – 4.43) | 1/5 (20%) | 1.12 (0.41 – 3.01) |
|  | *p=0.236* | *p=0.332* | *p=0.209* | *p=0.829* |
| Poor mental health |  |  |  |  |
| *No* | 65/120 (54%) | - | 45/70 (64%) | - |
| *Yes* | 10/34 (29%) | 0.35 (0.12 – 1.00) | 9/23 (39%) | 0.36 (0.13 – 0.96) |
|  | *p=0.011* | *p=0.051* | *p=0.034* | *p=0.041* |
| Communicates well with partner |  |  |  |  |
| *No* | 23/62 (37%) | - | 12/34 (35%) | - |
| *Yes* | 52/92 (57%) | 2.20 (1.04 – 4.68) | 42/59 (71%) | 4.53 (2.08 – 9.85) |
|  | *p=0.018* | *p=0.040* | *p=0.001* | *p<0.001* |
|  |  |  |  |  |
