# Supplementary material for: Longitudinal inconsistencies in women’s self-reports of lifetime experience of physical and sexual IPV: evidence from the MAISHA trial and follow-on study in North-western Tanzania
Source: BMC Womens Health. 2022 Apr 15;22:120. doi: 10.1186/s12905-022-01697-y (PMC9013096; doi:10.1186/s12905-022-01697-y)
Supplement: Supplementary file 3 — Additional file 3. Factors associated with discrepancies in IPV reporting between T0 and T53, excluding women with past year experience of the respective type of IPV at T53 (among women reporting ever having experienced each type of IPV at T0). Odds ratios (and 95% confidence intervals) of associations between baseline/T53 situational factors and discrepancies in IPV reporting between T0 and T53, excluding women with past year experience of the respective type of IPV at T53 (among women reporting ever having experienced each type of IPV at T0) [file 12905_2022_1697_MOESM3_ESM.docx]

Additional File 3: Factors associated with discrepancies in IPV reporting between T0 and T53, excluding women with past year experience of the respective type of IPV at T53 (among women reporting ever having experienced each type of IPV at T0)

| Baseline indicator | Physical  (n=133) | OR (95%CI) | Sexual  (n=72) | OR (95%CI) |
| --- | --- | --- | --- | --- |
| ***Discrepant reporting at T53*** | ***75/133 (56%)*** |  | ***54/72 (75%)*** |  |
|  |  |  |  |  |
| Age |  |  |  |  |
| *Under 35* | 25/44 (57%) | - | 23/28 (82%) | - |
| *35+* | 50/89 (56%) | 0.97 (0.41 – 2.29) | 31/44 (70%) | 0.52 (0.19 – 1.44) |
|  | *p=0.944* | *p=0.952* | *p=0.264* | *p=0.207* |
| Education |  |  |  |  |
| *Primary or below* | 57/100 (57%) | - | 37/51 (73%) | - |
| *Above primary* | 18/33 (55%) | 0.91 (0.54 – 1.51) | 17/21 (81%) | 1.61 (0.35 – 7.40) |
|  | *p=0.805* | *p=0.703* | *p=0.454* | *p=0.542* |
| Household-level financial hardship in past year |  |  |  |  |
| *No* | 45/69 (65%) | - | 26/30 (87%) | - |
| *Yes* | 30/64 (47%) | 0.47 (0.27 – 0.83) | 28/42 (67%) | 0.31 (0.10 – 0.99) |
|  | *p=0.033* | *p=0.009* | *p=0.053* | *p=0.049* |
| Past year experience of this type of IPV |  |  |  |  |
| *No* | 49/82 (60%) | - | 34/42 (81%) | - |
| *Yes* | 26/51 (51%) | 0.70 (0.30 – 1.62) | 20/30 (67%) | 0.47 (0.17 – 1.31) |
|  | *p=0.321* | *p=0.405* | *p=0.168* | *p=0.150* |
| Fear of partner in past year |  |  |  |  |
| *Never* | 60/93 (65%) | - | 37/45 (82%) | - |
| *A few times* | 9/21 (43%) | 0.41 (0.14 – 1.22) | 10/15 (67%) | 0.43 (0.11 – 1.64) |
| *Many/most/all of time* | 6/19 (32%) | 0.25 (0.09 – 0.70) | 7/12 (58%) | 0.30 (0.08 – 1.18) |
|  | *p=0.004* | *p=0.016* | *p=0.167* | *p=0.185* |
| Ever experience of one or more types of IPV (physical and/or sexual) |  |  |  |  |
| *One* | 49/72 (68%) | - | 16/19 (84%) | - |
| *Both* | 26/61 (43%) | 0.35 (0.17 – 0.70) | 38/53 (72%) | 0.48 (0.13 – 1.76) |
|  | *p=0.012* | *p=0.003* | *p=0.280* | *p=0.265* |
| Ever severe physical IPV |  |  |  |  |
| *No* | 25/41 (61%) | - | - | - |
| *Yes* | 50/92 (54%) | 0.76 (0.36 – 1.62) | - | - |
|  | *p=0.477* | *p=0.481* | *-* | *-* |
| Ever emotional IPV |  |  |  |  |
| *No* | 15/20 (75%) | - | 12/13 (92%) | - |
| *Yes* | 60/113 (53%) | 0.38 (0.16 – 0.91) | 42/59 (71%) | 0.21 (0.03 – 1.30) |
|  | *p=0.069* | *p=0.030* | *p=0.111* | *p=0.093* |
| **T53 situational variables** |  |  |  |  |
| Changed partnered since baseline |  |  |  |  |
| *No change/ left partner* | 58/108 (54%) | - | 39/53 (74%) | - |
| *New partner* | 17/25 (68%) | 1.83 (0.70 – 4.77) | 15/19 (79%) | 1.35 (0.36 – 5.08) |
|  | *p=0.194* | *p=0.215* | *p=0.643* | *p=0.661* |
| Poor mental health |  |  |  |  |
| *No* | 65/106 (61%) | - | 45/56 (80%) | - |
| *Yes* | 10/27 (37%) | 0.37 (0.13 – 1.07) | 9/16 (56%) | 0.31 (0.09 – 1.08) |
|  | *p=0.023* | *p=0.067* | *p=0.050* | *0.065* |
| Communicates well with partner |  |  |  |  |
| *No* | 23/50 (46%) | - | 12/20 (60%) | - |
| *Yes* | 52/83 (63%) | 1.97 (0.90 – 4.33) | 42/52 (81%) | 2.80 (1.02 – 7.72) |
|  | *p=0.061* | *p=0.092* | *p=0.068* | *p=0.047* |
|  |  |  |  |  |
